# New Human Papilloma Virus E2 Transcription Factor Mimics: A Tripyrrole-Peptide Conjugate with Tight and Specific DNA-Recognition

**DOI:** 10.1371/journal.pone.0022409

**Published:** 2011-07-25

**Authors:** Diana E. Wetzler, Maria J. Comin, Krzysztof Krajewski, Mariana Gallo

**Affiliations:** 1 Department of Biological Chemistry, FCEN, University of Buenos Aires, Buenos Aires, Argentina; 2 Center for Research and Development in Chemistry, INTI, Buenos Aires, Argentina; 3 Department of Biochemistry and Biophysics, School of Medicine, University of North Carolina, Chapel Hill, North Carolina, United States of America; 4 Fundación Instituto Leloir, Buenos Aires, Argentina; National Cancer Institute, United States of America

## Abstract

**Background:**

Human papillomavirus (HPV) is the main causative agent of cervical cancer, particularly high risk strains such us HPV-16, -18 and -31. The viral encoded E2 protein acts as a transcriptional modulator and exerts a key role in viral DNA replication. Thus, E2 constitutes an attractive target for developing antiviral agents. E2 is a homodimeric protein that interacts with the DNA target through an α-helix of each monomer. However, a peptide corresponding to the DNA recognition helix of HPV-16 E2 binds DNA with lower affinity than its full-length DNA binding domain. Therefore, in an attempt to promote the DNA binding of the isolated peptide, we have designed a conjugate compound of the E2 α-helix peptide and a derivative of the antibiotic distamycin, which involves simultaneous minor- and major-groove interactions.

**Methodology/Principal Findings:**

An E2 α-helix peptide-distamycin conjugate was designed and synthesized. It was characterized by NMR and CD spectroscopy, and its DNA binding properties were investigated by CD, DNA melting and gel shift experiments. The coupling of E2 peptide with distamycin does not affect its structural properties. The conjugate improves significantly the affinity of the peptide for specific DNA. In addition, stoichiometric amounts of specific DNA increase meaningfully the helical population of the peptide. The conjugate enhances the DNA binding constant 50-fold, maintaining its specificity.

**Conclusions/Significance:**

These results demonstrate that peptide-distamycin conjugates are a promising tool to obtain compounds that bind the E2 target DNA-sequences with remarkable affinity and suggest that a bipartite major/minor groove binding scaffold can be a useful approach for therapeutic treatment of HPV infection.

## Introduction

Cervical cancer is a major cause of cancer-related death in women. Human papillomavirus (HPV) is the main causative agent of cervical cancer, in particular high risk strains such us HPV-16 and -18 followed by HPV-31 and -45 [1]. Despite recent advances in preventive HPV vaccine development, such strategies are unlikely to reduce the prevalence of HPV infections within the next few years, due to their high cost and limited availability in developing countries. In addition, preventive HPV vaccines may not be capable of treating established HPV infections and HPV-associated lesions, which account for high morbidity and mortality worldwide. Thus, it is crucial to develop therapeutic strategies for controlling HPV infection and associated malignancies [2]. HPV E2 protein acts as a transcriptional modulator and participates in viral DNA replication and episomal copy number maintenance of the viral genome [3]. Furthermore, E2 represses the transcription of E6 and E7 oncoproteins, and this ability is lost upon viral DNA integration into the host genome. This event often occurs with the disruption of the E2 open reading frame, leading to the deregulation of the expression of E6 and E7, essential for the transformation process [4]. Hence, E2 constitutes an ideal target for HPV therapy. In particular, molecules capable of binding the HPV E2 DNA-recognition sequences with high specificity and affinity may interfere with one key event of the viral life cycle: the interaction between the E2 transcription factor and its target DNA sequences. It is expected that these molecules will act with similar efficiency on all the high-risk strains, as all these viruses have in common highly conserved E2 DNA binding sequences [5]. Furthermore, targeting a conserved DNA sequence, instead of a potentially mutable protein, can overcome the problem of resistant virus generation, a major drawback associated with antiviral agents.

There is a great deal of interest in obtaining small synthetic mimics of transcription factors capable of reproducing their DNA-binding properties but also presenting good bioavailability [6], [7]. E2 consists of an N-terminal transactivation domain and a C-terminal DNA binding and dimerization domains (E2C), separated by a nonconserved hinge domain [8]. E2C is a symmetric dimer that interacts with the major groove of the target DNA through two α-helices, one of each monomer [8], [9], [10]. A peptide corresponding to the DNA recognition helix of HPV-16 E2 displays only residual structure and binds DNA with low affinity [11], [12]. Recently, it was demonstrated in various biological systems that the appropriate tethering of a monomeric DNA-binding helix to a distamycin (*Dst*)-like tripyrrole, which binds with moderate affinity the minor groove adjacent to the helix target site, provides for specific binding [13], [14], [15]. The natural cognate DNAs of HPV-16 and -18 E2 present, in the region adjacent to the DNA-sequence recognized by E2 (CGGT), the sequence AAAT, which is one of the sequences with the highest binding affinities for *Dst* [16]. Therefore, we decided to explore whether conjugation of a peptide corresponding to the DNA-binding α-helix of the E2 protein from HPV-16 with a *Dst* derivative could improve the affinity of the peptide for specific DNA. Here we report the design, synthesis, characterization and DNA-binding properties of a conjugate between a peptide corresponding to the α-helix of the HPV-16 E2 protein and a *Dst* tripyrrole derivative carrying a primary amino function in the tether between both moieties. These results show the potential utility of this kind of hybrids as a new class of molecules for the treatment of HPV infections and are encouraging for the design of E2 conjugates with alternative linkers that can bind DNA with higher specificity and affinity.

## Materials and Methods

**Figure 1:**
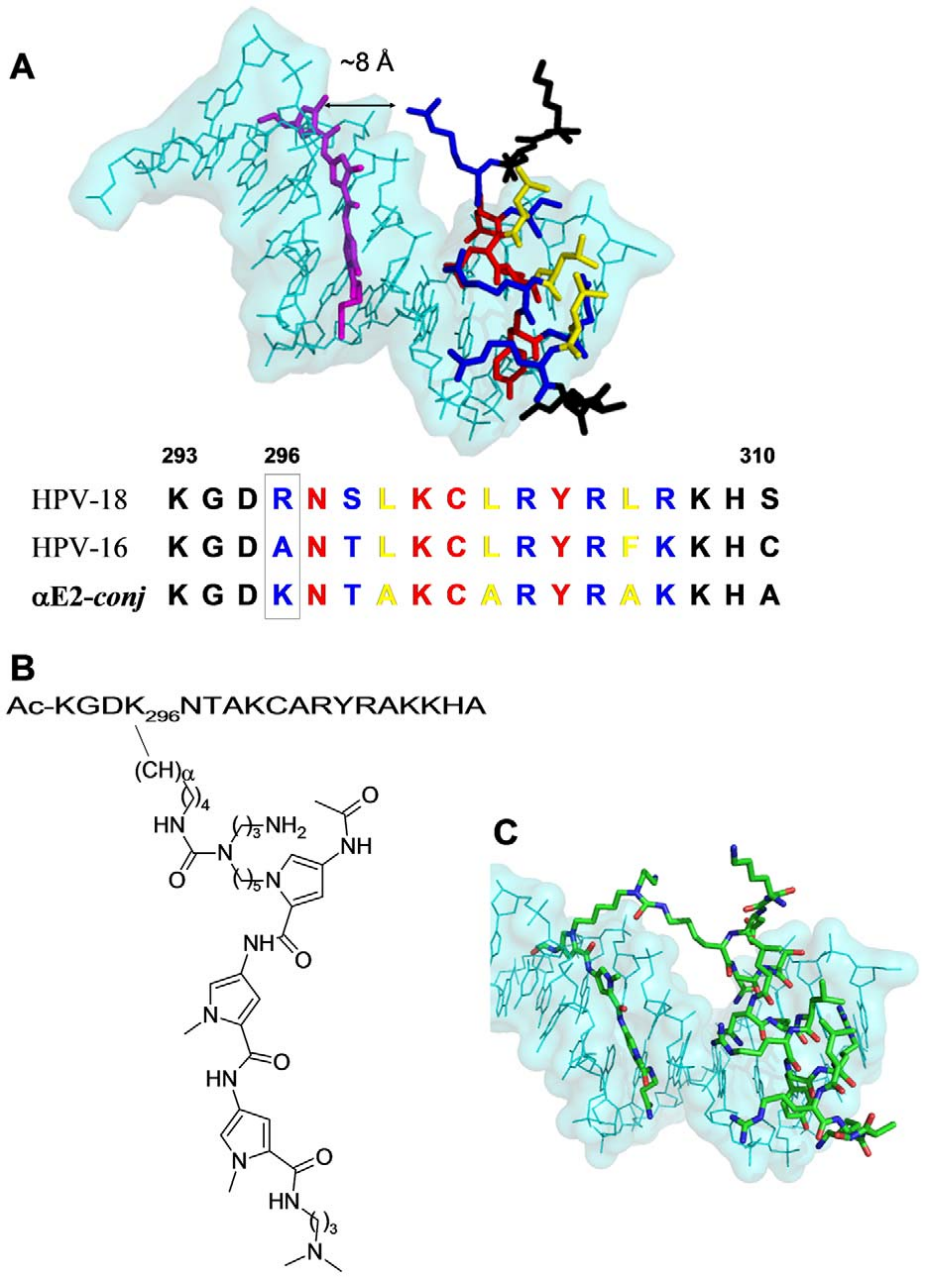
Design of αE2-*conj*. **A. Hypothetic model for the simultaneous binding of the αE2 peptide and** ***Dst*** **to DNA**. DNA is depicted in cyan and *Dst* in magenta. The peptide positions involved in backbone DNA recognition are displayed in blue; in red are shown residues involved in specific DNA bases interactions; while in yellow are the hydrophobic residues mutated to alanine to minimize hydrophobic interactions. The distance between the N2 of *Dst* and the N-atoms of the guanidinium group of R296 is indicated. The lower panel shows the alignment of the DNA binding helices of E2 proteins from HPV-16 and -18 strains, and the sequence of the peptide used for synthesizing **αE2-** ***conj***. The colour coding is the same used in the model. Position 296 chosen to attach the *Dst* moiety is boxed. **B. Chemical structure of αE2-** ***conj***. **C. Qualitative model of αE2-** ***conj*** **bound to DNA**.

In this study we have worked with the following compounds: **αE2**: peptide corresponding to the DNA-binding α-helix of the E2 protein from HPV-16; **αE2** ***-Ala***: the **αE2** peptide but with alanine in positions 299, 302 and 306; **αE2** ***-conj***: the **αE2** ***-Ala*** peptide *Dst* conjugate (see **Figure 1B** below).

### Computer modelling

The model for the simultaneous interaction of *Dst* and a peptide corresponding to the DNA-binding α-helix of the E2 protein with DNA was constructed using the program Insight II and was based on the crystal structures of the complexes of E2C from HPV-18 (PDB 1F9F) [5] and *Dst* (PDB 1JTL) [17] with their cognates DNAs. Both structures were superimposed by phosphate alignment to get an appropriate arrangement for connecting the two moieties. The linker was designed by using the module Builder of this program. We considered only one of the two possible binding modes of *Dst* to DNA for designing the conjugate [17].

### Synthesis of αE2-*conj*

Linear peptides were synthesized by an automated SPPS (ABI 433A Peptide Synthesizer) using Fmoc chemistry (coupling with HBTU/HOBt/DIPEA in NMP). Purification of the crude conjugate was performed by preparative RP-HPLC (Vydac C18 column). Purified products were further characterized by Kompact Axima-CFR MALDI-TOF mass spectrometer (see Text S1).

### DNA Synthesis

Single-stranded oligonucleotides were purchased, SDS-PAGE purified, from Primm srl (Milano, Italy). Double-stranded oligonucleotides were annealed by mixing equal amounts of each strand in 10 mM Bis-Tris-HCl buffer, pH 7.0 and 50 mM NaCl, incubating 5 minutes at 95°C, and slowly cooling to 25°C for 16 hours. Completeness of hybridization was checked by native polyacrylamide gel electrophoresis.

### NMR Spectrosocopy

The conjugate **αE2-** ***conj*** (3 mM) and the corresponding isolated peptide, **αE2-** ***Ala*** (KGDANTAKCARYRAKKHA), (7 mM) were dissolved in a 40% TFE-*d*3 - 60% 20 mM phosphate buffer solution pH 6.5 containing 2 mM DTT and 0.01% NaN_3_. The NMR experiments were performed at 25°C using a Bruker Avance 700 and Avance 400 spectrometers equipped with triple resonance probes incorporating self-shielded gradient coils. All the heteronuclear correlation experiments were carried out at natural abundance. Pulsed field gradients were appropriately employed to achieve suppression of the solvent signal and spectral artefacts. Quadrature detection in the indirectly detected dimensions was obtained using the States-TPPI method [18] or the echo-antiecho method, and the spectra were processed on Silicon Graphics workstations by the NMRPipe software [19] and analyzed using NMRView [20]. All proton, nitrogen and carbon resonances were assigned using 2D spectra. TOCSY (mixing time 60 ms) was used to identify the spin systems. Natural abundance [^1^H-^13^C] HMQC optimized for observing aliphatic or aromatic regions and [^1^H-^13^C] HMQC-TOCSY (with mixing times of 40, 60 and 80 ms) spectra were used to assist with cross-peaks assignment. NOESY spectra (with mixing times of 0.150 s and 0.250 s) were used for establishing the sequential assignment of the spin systems. ^15^N chemical shifts were measured from a natural abundance [^15^N-^1^H] HSQC experiment.

### CD Spectroscopy

CD measurements were carried out on a Jasco J-810 spectropolarimeter using a Peltier temperature-controlled sample holder and a 0.1 cm path length cell. Near UV experiments were performed with 2 µM hemisite E2-*Dst* site alone and mixtures containing 10 µM **αE2-** ***conj*** or *Dst* at 25°C. Far UV spectra and *K_D_* determination experiments were performed at 5°C in solutions containing 5 µM peptide after incubating 20 minutes at room temperature with a stoichiometric amount of the corresponding hemisite.

CD titration experiments were done with 5 µM **αE2-conj** and increasing concentrations of hemisite E2-*Dst*. The change in helical population was detected following the ellipticity change at 222 nm and fitted to a 1∶1 binding model using the following equation:
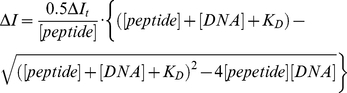
where Δ*I* is the signal change at each DNA concentration, Δ*I*_t_ is the signal difference between the free peptide and the complex, *K_D_* is the equilibrium dissociation constant and [peptide] and [DNA] are the respective total concentrations.

All CD experiments were done in 10 mM phosphate pH 7.0, 1 mM DTT, 100 mM NaCl buffer.

### DNA melting experiments

UV DNA melting experiments were conduced with the oligonucleotide (2 µM) in the absence and in the presence of an excess of peptide (10 µM) in the same buffer conditions used in CD experiments. Absorbance at 260 nm was measured as a function of temperature using the absorbance mode of the Jasco spectropolarimeter with its Peltier temperature-controlled sample holder. The melting temperature (Tm) was determined as the midpoint of the curve. Each Tm is the mean value obtained from three independent measurements.

### Electrophoretic Mobility Shift Assay (EMSA)

Reaction mixtures contained 2 µM fluorescein-labelled oligonucleotides and increasing amounts of the studied peptides. Mixtures were incubated 60 min at room temperature in a final volume of 10 µL in 25 mM phosphate pH 7.0, 1 mM DTT. Mixtures were loaded continuously into running non-denaturing 10% polyacrylamide gels containing TBE 0.5× (0.1 M Tris-HCl pH 8.0, 0.15 M sodium borate, 4 mM EDTA). The gels were resolved at 4 V/cm, 10°C for 2 h. Fluorescein bands corresponding to the free oligonucleotide were detected by UV and fitted to a 1∶1 binding model (the same equation than in the CD titration but with a fixed concentration of peptide and increasing concentrations of DNA). Because of the peptide's high isoelectric point (pI∼11), free DNAs and αE2-DNA complex migrate in opposite directions [11], so the complex band was not observed in the gels and only free DNA was quantified in the experiments.

## Results and Discussion

### Design of the conjugate αE2-*conj*

In order to design our conjugate, we constructed a model for the simultaneous interaction of *Dst* and the E2 helix peptide with adjacent DNA sites based on the structures of the complexes of the HPV-18 E2C domain [5] and *Dst* [17] with their cognates DNAs (**Figure 1A**). First, we changed hydrophobic residues in the peptide, which are not involved in DNA recognition, into alanines to reduce unfavourable hydrophobic interactions, to augment the water solubility, and possibly to increase the helix tendency of the peptide [21]. Second, side chain of R296 is very close to the N2 of *Dst* (∼8 Å between N2 of *Dst* and the N-atoms of the guanidinium group of R296). Therefore, these positions were chosen for tethering the peptide and the tripyrrole derivative. As R296 is not involved in the interaction with DNA bases [10], [22] and, since lysine was successfully used to attach linkers between peptides and tripyrrole units [14], [23], [24], we introduced in position 296 a lysine. Finally, the selection of the linker is crucial to have high affinities hybrids [14], [15]. We designed a *Dst*-peptide conjugate, named **αE2-** ***conj*** (**Figure 1B**). In this compound, the *Dst* bound to a pentylamine is connected to the nitrogen-ζ of the side chain of the K296 by a urea functionality [14]. According to our model, this linker length with a pentylene between the urea and the pyrrolic nitrogens should be adequate to allow the simultaneous binding of both moieties to DNA, the *Dst* in the minor groove and the peptide in the major groove (**Figure 1C**). The additional primary propylamine, which is protonated at the working pH, may also favour the affinity towards the negatively charged nucleic acid [15].

### Synthesis and NMR characterization of αE2-*conj*

The tripyrrole unit was prepared following reported procedures [13]. Selective functionalization at K296, required to attach the minor groove binding moiety, was performed through the introduction of an Alloc-protected side chain lysine residue [23]. Removal of the Alloc-protecting group and attachment of the tripyrrole unit was performed with the protected peptide still bound to the resin. Coupling reaction was carried out by sequential addition of the bifunctional conjugating agent disuccinimidyl carbonate and the amino pyrrole. After standard cleavage-deprotection and purification steps, we obtained the desired conjugated **αE2-** ***conj*** in approximately 20% yield considering peptide synthesis (see Text S1).

**Figure 2:**
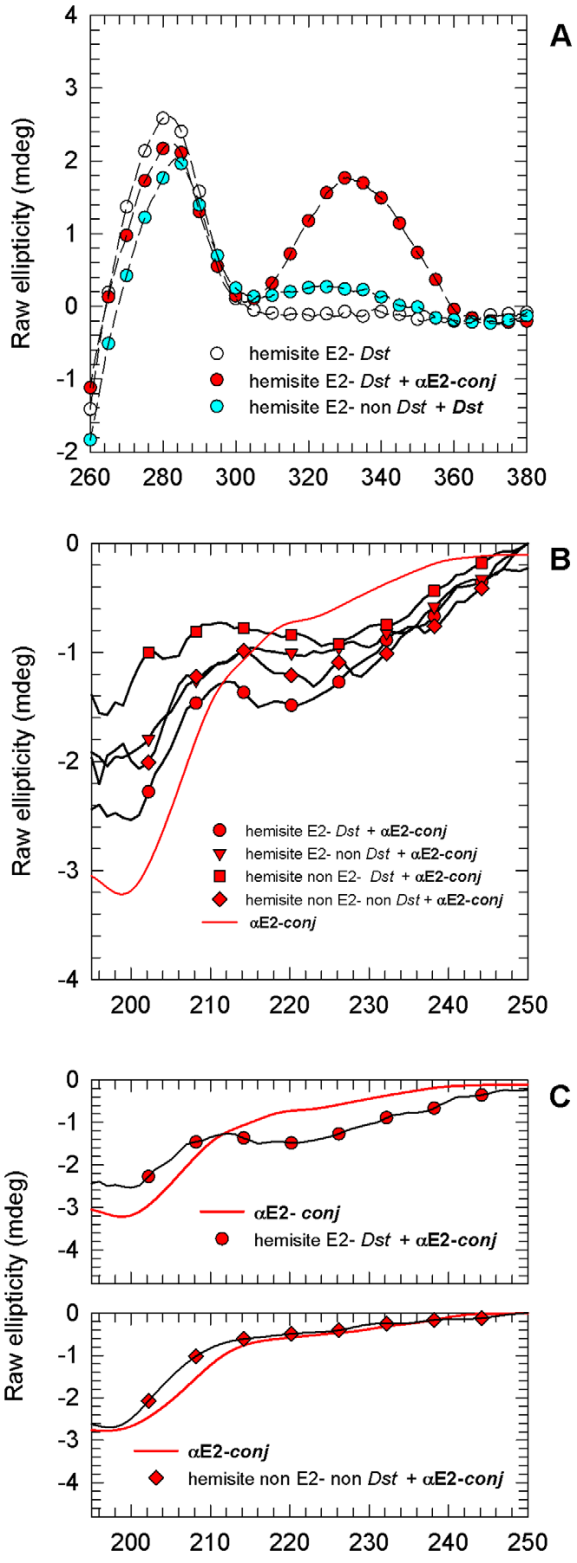
Spectroscopic interaction evidence of αE2-*conj* and DNA. **A.** Near-UV CD spectrum at 25°C of the oligonucleotide hemisite E2-*Dst* (2 µM) alone, after the addition of 10 µM *Dst* or **αE2-** ***conj***. **B.** Far-UV CD spectrum of **αE2-** ***conj*** alone, in the presence of hemisite E2-*Dst*, hemisite E2-non *Dst*, hemisite non E2-*Dst* or hemisite non E2-non *Dst*. **C.** Far-UV CD spectrum of **αE2-** ***conj*** alone and in the presence of hemisite E2-*Dst* (upper panel), and of **αE2-** ***Ala*** alone and in the presence of hemisite E2-*Dst* (lower panel). CD measurements in **B** and **C** were performed at 5°C in solutions containing 10 mM phosphate buffer pH 7.0, 100 mM NaCl, and 5 µM peptide after incubating 20 minutes at room temperature with 5 µM ds-oligonucleotide, when present. **B** and **C** show the spectrum of the mixture (peptide and DNA) minus the spectra of the corresponding DNA alone.

**αE2-** ***conj*** was characterized by NMR spectroscopy (see Text S2, **Figure S1** and **Tables S1** and **S2**). The coupling with the *Dst* moiety does not affect significantly the conformational properties of the peptide moiety respect to the isolated peptide (**Figure S1C**). Analysis of the secondary chemical shifts in the **αE2-** ***conj*** peptide indicates that in a 4∶6 TFE-water solution it adopts an α-helical conformation from K296 to H309 (**Figure S2**). In contrast, in aqueos solution the peptide moiety is mainly unstructured as shown by CD spectroscopy (**Figure 2B**). Additionally, the secondary chemical shift analysis shows that the α-helix population is mainly the same as that found for the isolated HPV-16 E2 helix peptide, **αE2** (**Figure S2**). This result points out that the substitution of three hydrophobic residues for alanines was not sufficient for augmenting the helix propensity of the peptide, at least in the TFE: water mixture. Similar helical propensity of **αE2**, **αE2-** ***conj*** and **αE2-** ***Ala*** was confirmed by CD spectroscopy in TFE titration experiments following molar ellipticty at 222 nm (Text S3, **Figure S3**).

### DNA binding properties of αE2-*conj*

A first evidence of the interaction of **αE2-** ***conj*** with DNA was obtained from CD spectroscopy (**Figure 2**, and see **Table 1** for sequences of oligonucleotides used in this work). The appearance of an intense positive band around ∼330 nm is observed in the spectrum of the oligonucleotide hemisite E2*-Dst* upon addition of an excess of **αE2-** ***conj*** (**Figure 2A**). This is an indication of the tripyrrole moiety of **αE2-** ***conj*** inserting into the DNA minor groove. The fact that only a slight increase in the ellipticity at 330 nm is induced in the spectrum of hemisite E2-*Dst* after adding *Dst* shows that both moieties of **αE2-** ***conj*** must be present for a tight binding to DNA. On the other hand, the far-UV CD spectrum of **αE2-** ***conj*** in aqueous solution corresponds to that of a mostly unfolded peptide (**Figure 2B**). However, a significant increase in the negative CD signal at 222 nm in the spectrum of **αE2-** ***conj*** was induced when added hemisite E2*-Dst*, indicating the folding in α-helix of the peptide moiety upon DNA binding. Control oligonucleotides, lacking the E2 or the *Dst* recognition sequences, or both, produced a minor increase of the 222 nm band, suggesting some nonspecific but weaker binding. Additionally, the CD signal at 222 nm is not significantly augmented in the spectrum of the isolated peptide **αE2-** ***Ala*** when incubated with hemisite E2-*Dst* oligonucleotide (**Figure C**, lower panel). These results demonstrate that interaction of both peptide and *Dst* are necessary in **αE2-** ***conj*** for a tight binding.

**Table 1:** Sequences of duplex oligonucleotides used in this study.

Oligonucleotides^a^	Sequence^a^
hemisite E2*-Dst* b	GTA GCA C***aa at*** **C GGT** TGA
	TCA **ACC G** ***at tt***G TGC TAC
hemisite E2-non *Dst*	GTA GCA CAG CT**C GGT** TGA
	TCA **ACC G**AG CTG TGC TAC
hemisite non E2*-Dst*	GTA GCA C***aa at***T GCG TGA
	TCA CGC A***at tt***G TGC TAC
hemisite non E2-non *Dst*	GTA GCA C*AG* CTT GCG TGA
	TCA CGC AAG CTG TGC TAC
Iset non E2-non *Dst*	ACA TGG ACC TGT CAA GTA
	TAC TTG ACA GGT CCA TGT

a The E2 hemisite is underlined and the *Dst* binding site is indicated in italics and in lower-case letters.

b The hemisite E2*-Dst* corresponds to half E2 target site 35.

**Figure 3:**
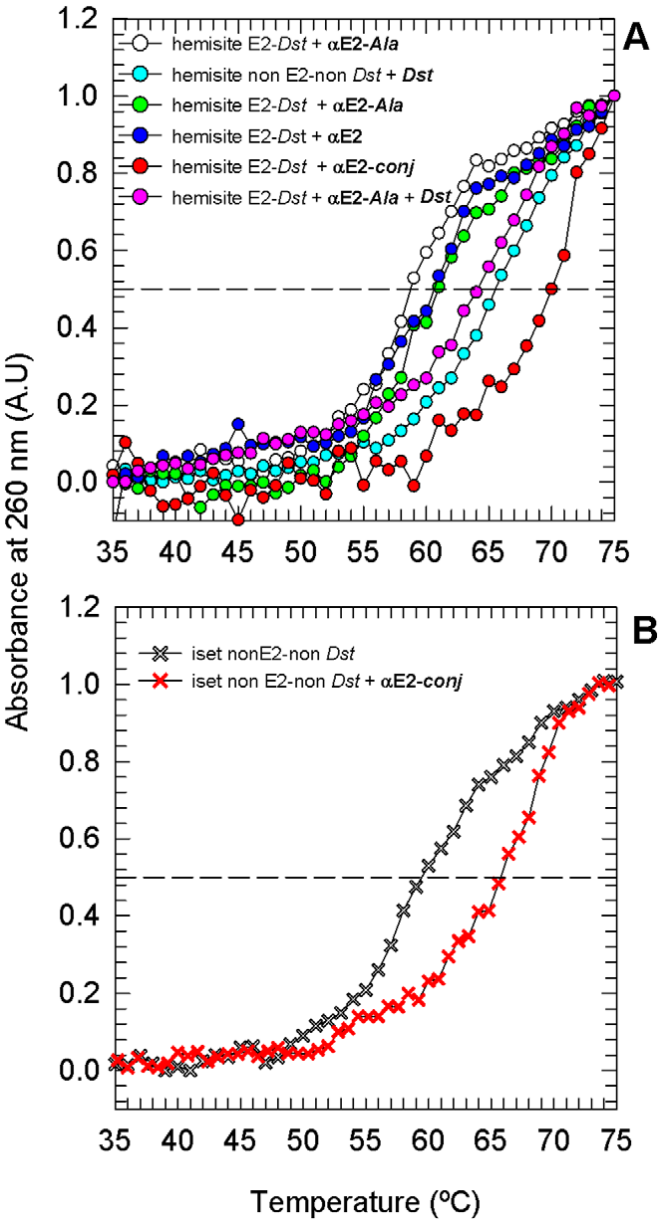
DNA melting curves. **A**. Melting curves of the oligonucleotide hemisite E2- *Dst* alone, in the presence of **αE2**, **αE2-** ***Ala***, *Dst* plus **αE2-** ***Ala***, *Dst* and **αE2-** ***conj***. **B**. Melting curves of the oligonucleotide Iset non E2-non *Dst* alone and in the presence of **αE2-** ***conj***. Table **2** summarizes these results.

Supplementary evidence of specific binding of **αE2-** ***conj*** to DNA was obtained by ds-DNA melting temperature (Tm) measurements (**Figure 3** and **Table 2**). When some compound binds ds-DNA, the duplex is stabilized by the interaction and the Tm of the nucleic acid is increased. Tighter the binging is, higher the melting temperature. The oligonucleotide hemisite E2-*Dst* melts at 58.5°C (**Figure 3A**). In the presence **αE2** or **αE2-** ***Ala*** the melting is slightly augmented, due to the weak interaction of the isolated peptides with specific DNA [11]. In contrast, in the presence of *Dst* the increase in the melting temperature of hemisite E2-*Dst* is higher (7.0°C vs ∼2°C). This augment reflects the tripyrrole binding in the DNA minor groove, as was observed in the CD measurements. Finally, when **αE2-** ***conj*** is added the Tm is the highest measured (70.0°C), showing that a tight binding is obtained only when both moieties of **αE2-** ***conj*** are present. Interestingly, the Tm in the simultaneous presence of **αE2-** ***Ala*** and *Dst* was even lower than in the presence of only *Dst*, indicating that the effect is not additive. On the other hand, **αE2-** ***conj*** produces a higher increase in the Tm of the hemisite E2-*Dst* than in the Tm of two ds-oligonucleotides not containing the E2 nor the Dst binding sites: hemisite non E2-non *Dst* and Iset non E2-non *Dst* (ΔTm of 6.0°C for both control oligonucleotides vs. 11.5°C for hemisite E2-*Dst*, **Figure 3B** **and** **Table 2**), suggesting that the binding of **αE2-** ***conj*** is specific.

**Table 2:** DNA melting temperatures.

hemisite E2-*Dst*		
	Tm (°C)^a^	ΔTm (°C)
alone	58.5±0.3	–
+*Dst*	65.5±0.4	7.0±0.7
+**αE2-** ***Ala***	61.0±0.3	2.5±0.6
+**αE2**	60.5±0.5	2.0±0.8
+**αE2-** ***conj***	70.0±0.3	11.5±0.6
+**αE2-** ***Ala*** +*Dst*	64.5±0.4	6.0±0.7

hemisite non E2-non *Dst*		
alone	64.4±0.4	–
+**αE2-** ***conj***	70.0±0.4	6.0±0.8

Iset non E2-non *Dst*		
alone	60.2±0.2	
+**αE2-** ***conj***	66.2±0.3	6.0±0.5

a mean values ± standard deviation between three independent measurements.

**Figure 4:**
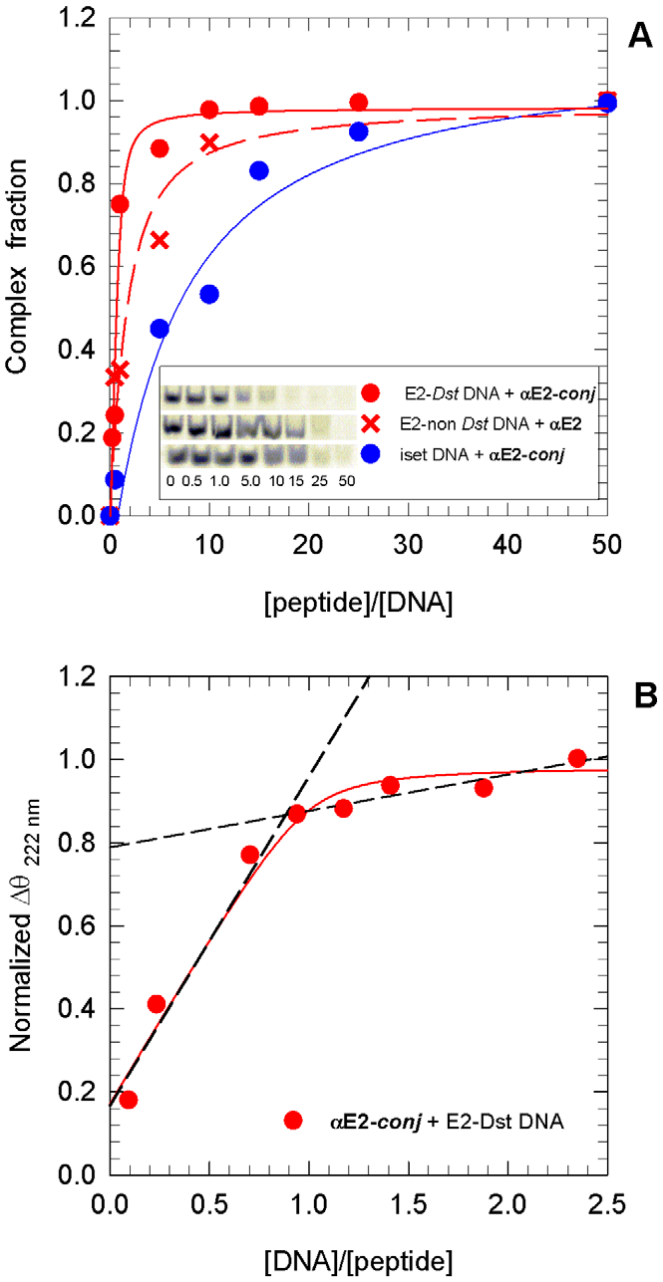
Equilibrium dissociation constant determination. **A.** Electrophoretic mobility shift assay at room temperature, complex fraction as a function of peptide/DNA ratio: **αE2-** ***conj***/hemisite E2-*Dst*, **αE2**/hemisite E2-*Dst*, **αE2-** ***conj***/Iset non E2-non *Dst*. The inset shows a picture of the corresponding gel assays. **B.** Molar ellipticity change at 222 nm as a function of hemisite E2-*Dst/* **αE2-** ***conj*** ratio, experiment performed at 5°C. Both titration experiments were carried out in 10 mM phosphate pH 7.0, 100 mM NaCl, 1 mM DTT buffer. Lines are fits to a quadratic equation corresponding to 1∶1 binding model. Dash lines in figure **B** are added to show 1∶1 stoichiometry.

The full E2C domain in solution binds DNA with high affinity (*K_D_* in the low nanomolar range when determined in solution) and specificity (∼1000 fold) [9], [25]. However, when the affinity was measured by gel shift experiments, the *K_D_* for a complex between E2C and a specific ds-oligonucleotide was in the µM order [9]. Dissociation constants of **αE2-** ***conj*** and **αE2** to specific and unspecific oligonucleotides at 25°C were determined by gel shift experiments (**Table 3** and **Figure 4A**). The complex between the **αE2** peptide and hemisite E2-*Dst* gave a *K_D_* of ∼6 µM, in agreement with previous measurements [11], [12]. The *K_D_* for a complex between **αE2** and the randomnized oligonucleotide Iset non E2-non *Dst* was of ∼50 µM, also in perfect agreement with previous measurements. On the contrary, the complex between the conjugate **αE2-** ***conj*** and hemisite E2-*Dst* presented a *K_D_* of ∼0.13 µM. Thus **αE2-** ***conj*** displays an increment of affinity for the target DNA of one order and a half (*K_D_* (**αE2**)/*K_D_* (**αE2-** ***conj***) = 48). Regarding E2C, **αE2-** ***conj*** exhibits an augment of affinity for specific DNA of one order of magnitude, when measured in the same conditions [9]. The specificities of **αE2** and **αE2-** ***conj*** are similar (*K_D_* (Iset non E2-non *Dst*)/*K_D_* (Iset non E2-non *Dst*) is 8 and 9 for **αE2** and **αE2-** ***conj***, respectively).

**Table 3:** Dissociation constants.

Complex	*K_D_* (µM)	
	gel shift experiments	CD titration
**αE2**/hemisite E2-*Dst*	6.3±2.6	*n.d.* a
**αE2/**Iset non E2-non *Dst*	∼50	*n.d.*
**αE2-** ***conj***/hemisite E2-*Dst*	0.13±0.09	0.10±0.15
**αE2-** ***conj*** **/**Iset non E2-non *Dst*	1.22±0.75	*n.d.*
**E2C**/hemisite E2-*Dst*	∼1^b^	*n.d.*

a *n.d.*: not determined.

b Ref. 9.

The *K_D_* of **αE2-** ***conj*** hemisite E2-*Dst* complex was also determined by CD titration at 222 nm at 5°C (0.10±0.15 µM, **Table 2** and **Figure 4B**). As expected, the value thus obtained is slightly lower than the value obtained by gel shift at 25°C. The observed differences are not related with the incubation buffer conditions but with dissociating conditions of the EMSA assay [26]. Moreover, the value might be overestimated because this measurement was performed at high concentrations to stress the helical signal in the CD spectrum. The CD experiment additionally shows that the stoichiometry of the complex is 1∶1.

### Conclusions

In this study we have demonstrated that it is possible, by properly conjugating the peptide to a *Dst*-like tripyrrole unit, to increase the affinity of the monomeric **αE2** peptide for specific binding to its cognate DNA. We have designed a conjugate carrying a primary amino function in the tether linking the peptide and the *Dst* moieties to facilitate interaction with the phosphate DNA-backbone. Our conjugate displays a ∼50-fold increase of the affinity for specific DNA respect to the peptide alone and a ∼10-fold increase when compared with the full-length domain, also measured by gel shift experiments [9]. These results are encouraging for the design of new E2 conjugates with alternative linkers with higher specificities and better affinities capable of acting as antiviral agents in HPV infections.

Work to design new more potent conjugates and to structurally characterize them is currently in progress, in order to possibly further understand the factors that govern sequence-specific protein-DNA interactions.

## Supporting Information

Figure S1 **NMR chemical shift assignments of αE2-** ***conj*** **.** **A. Chemical structure and numbering of αE2-** ***conj*** **.** Inset: An alternative chemical connection between the peptide and the *Dst* derivative moieties. **B. ^1^H-monodimensional spectrum of αE2-** ***conj*** **.** **C. [^13^C,^1^H]-HMQC spectra of αE2-** ***Ala*** **and αE2-** ***conj*** (right and left panels, respectively). The spectra were recorded in a 700 MHz spectrometer at 25°C in 4∶6 TFE∶aqueous solution. Residue chemical shift assignment of the peptide moiety is indicated in black and signals corresponding to the linker and *Dst* portions of the conjugate are indicated in red. In bold letters are shown the crosspeaks corresponding to residue in position 296 in both compounds, for the isolated peptide 296 is an alanine while is a lysine in the conjugate. (Imp: Impurity).
(TIF)

Figure S2 **Secondary chemical shifts for αE2-** ***conj*** **and αE2.** Plot of the chemical shift differences between the observed resonances and values found in a random coil conformation, Δδ = δ(obsd) - δ(random coil), vs. the position along the peptidic sequence for ^13^C_α_, ^13^C_β_ and ^1^H_α_ on the top, medium, and lower panel, respectively, for **αE2-** ***conj*** (•) and **αE2** (○). Positive deviations of the shifts of C_α_ and negative of C_β_ and H_α_ are indicative of α-helical conformation.
(TIF)

Figure S3 **TFE titration for αE2-** ***Ala*** **, αE2 and αE2-** ***conj*** **.** Molar ellipticity at 222 nm for **αE2-** ***Ala***, **αE2**, **αE2-** ***conj*** as a function of TFE concentration. Line corresponds to the fit to a two-state coil-helix equilibrium proposed.
(TIF)

Table S1 **^1^H, ^13^C and ^15^N Chemical shifts assignments of the peptide moiety of αE2-** ***conj*** **.**
(DOCX)

Table S2 **^1^H, ^13^C, and ^15^N chemical shifts assignments of the** ***Dst*** **moiety of αE2-** ***conj*** **.**
(DOCX)

Text S1 **αE2-** ***conj*** **purification.**
(DOC)

Text S2 **NMR chemical shit assignments of αE2-** ***conj*** **.**
(DOC)

Text S3 **TFE titrations.**
(DOC)
