# Supplementary figures and images for: New Human Papilloma Virus E2 Transcription Factor Mimics: A Tripyrrole-Peptide Conjugate with Tight and Specific DNA-Recognition
Source: PLoS One. 2011 Jul 25;6(7):e22409. doi: 10.1371/journal.pone.0022409 (PMC3143144; doi:10.1371/journal.pone.0022409)

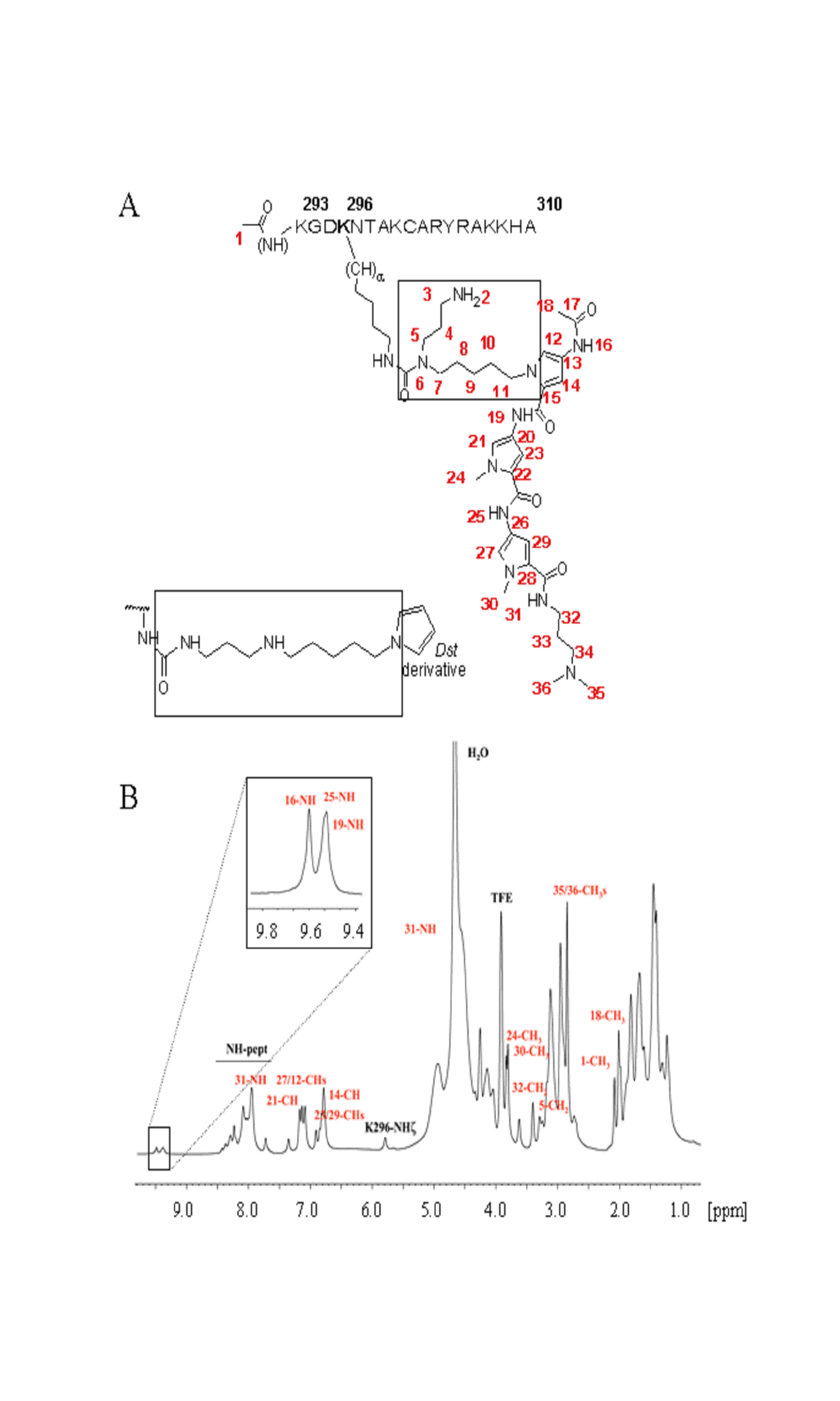

Supplement: Figure S1 — NMR chemical shift assignments of αE2- conj . A. Chemical structure and numbering of αE2- conj . Inset: An alternative chemical connection between the peptide and the Dst derivative moieties. B. 1H-monodimensional spectrum of αE2- conj . C. [13C,1H]-HMQC spectra of αE2- Ala and αE2- conj (right and left panels, respectively). The spectra were recorded in a 700 MHz spectrometer at 25°C in 4∶6 TFE∶aqueous solution. Residue chemical shift assignment of the peptide moiety is indicated in black and signals corresponding to the linker and Dst portions of the conjugate are indicated in red. In bold letters are shown the crosspeaks corresponding to residue in position 296 in both compounds, for the isolated peptide 296 is an alanine while is a lysine in the conjugate. (Imp: Impurity). (TIF) [file pone.0022409.s001.tif]

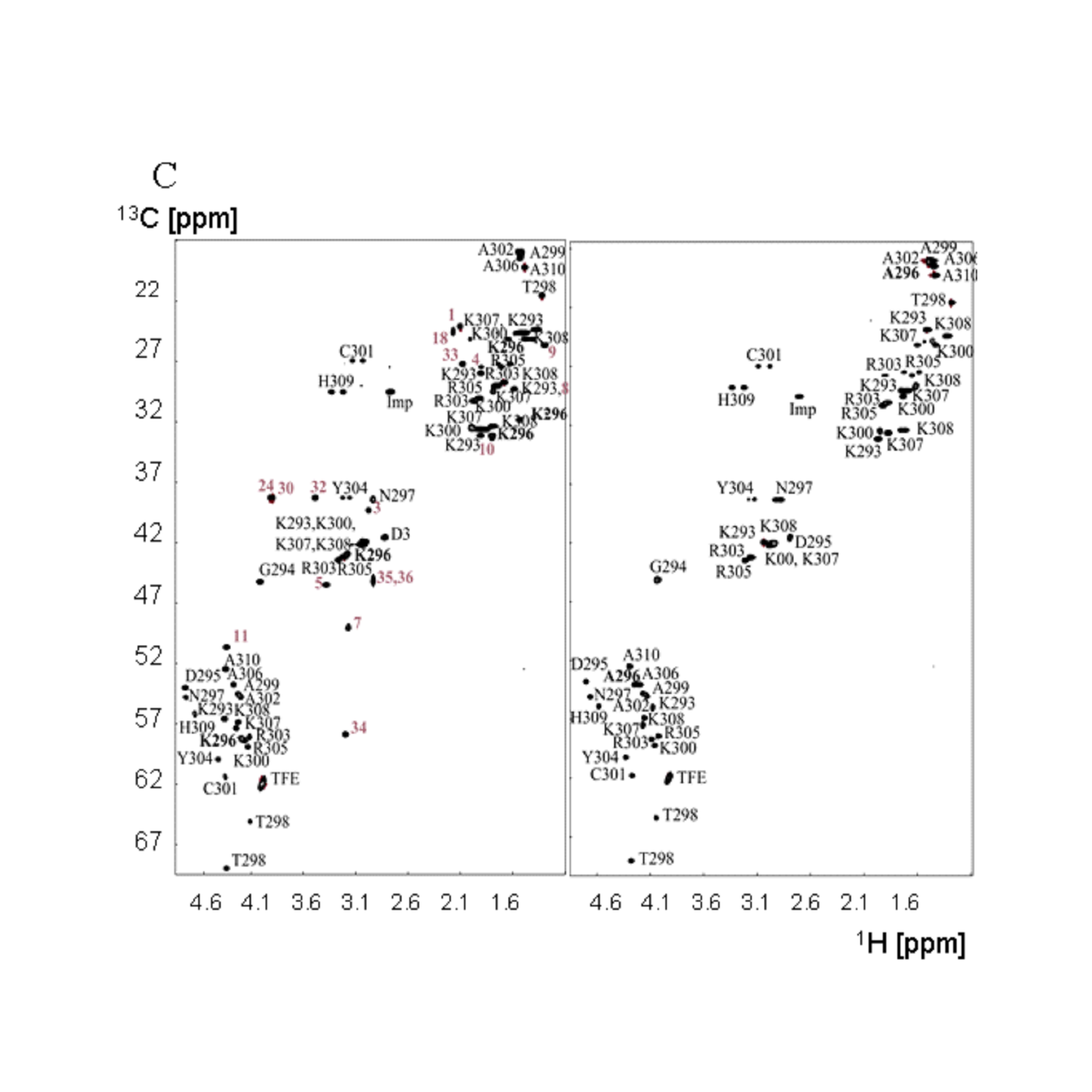

Supplement: Figure S2 — Secondary chemical shifts for αE2- conj and αE2. Plot of the chemical shift differences between the observed resonances and values found in a random coil conformation, Δδ = δ(obsd) - δ(random coil), vs. the position along the peptidic sequence for 13Cα, 13Cβ and 1Hα on the top, medium, and lower panel, respectively, for αE2- conj (•) and αE2 (○). Positive deviations of the shifts of Cα and negative of Cβ and Hα are indicative of α-helical conformation. (TIF) [file pone.0022409.s002.tif]

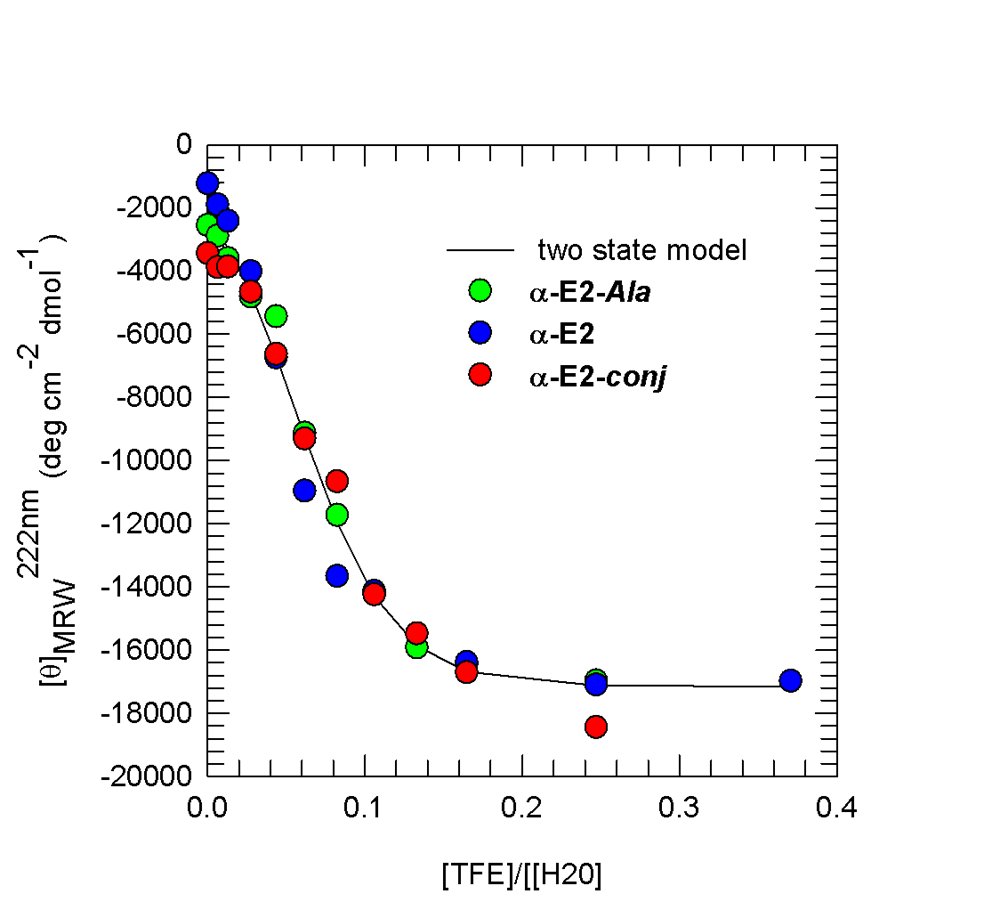

Supplement: Figure S3 — TFE titration for αE2- Ala , αE2 and αE2- conj . Molar ellipticity at 222 nm for αE2- Ala, αE2, αE2- conj as a function of TFE concentration. Line corresponds to the fit to a two-state coil-helix equilibrium proposed. (TIF) [file pone.0022409.s003.tif]
