# Supplementary material for: New Human Papilloma Virus E2 Transcription Factor Mimics: A Tripyrrole-Peptide Conjugate with Tight and Specific DNA-Recognition
Source: PLoS One. 2011 Jul 25;6(7):e22409. doi: 10.1371/journal.pone.0022409 (PMC3143144; doi:10.1371/journal.pone.0022409)
Supplement: Table S1 — 1H, 13C and 15N Chemical shifts assignments of the peptide moiety of αE2- conj . (DOCX) [file pone.0022409.s004.docx]

**Table S1.** ^1^H, ^13^C and ^15^N Chemical shifts assignments of the peptide moiety of **αE2-*conj*** in a 4:6 TFE:aqueous solution (20 mM phosphate buffer pH 6.5, 2 mM DTT and 0.01% NaN_3_) at 25 °C and pH 6.5.^a^

| Residue | ^15^N | NH | ^13^C_α_ | ^1^H_α_ | ^13^C_β_ | ^1^H_β_ | Others^b^ |
| --- | --- | --- | --- | --- | --- | --- | --- |
| **K293** | 125.5 | 8.08 | 56.6 | 4.29 | 33.2 | 1.84/1.73 | γ 24.2 (1.46/1.46), δ 29.3 (1.51/1.51), ε 42.4 (3.00/3.00) |
| **G294** | 108.4 | 8.30 | 45.2 | 3.95/3.95 |  |  |  |
| **D295** | 119.6 | 8.09 | 54.0 | 4.67 | 41.6 | 2.76/2.76 |  |
| **K296** | 121.5 | 8.29 | 58.2 | 4.13 | 32.8 | 1.82/1.82 | γ 25.1 (1.34/1.42), δ 31.8 (1.47/1.47), ε 42.9 (3.12/3.12), ζ NH (5.80) |
| **N297** | 117.3 | 8.38 | 54.8 | 4.66 | 38.5 | 2.87/2.87 | δ NH 112.0 (7.73/6.78) |
| **T298** | 115.2 | 8.09 | 65.1 | 4.05 | 69.0 | 4.27 | γ 21.4 (1.26) |
| **A299** | 123.8 | 8.01 | 54.5 | 4.16 | 17.9 | 1.46 |  |
| **K300** | 118.4^c^ | 7.95 | 58.9 | 4.07 | 32.5 | 1.93/1.93 | γ 25.0 (1.41/1.58), δ 29.5 (1.72/1.72), ε 42.1 (2.97/2.97) |
| **C301** | 116.9 | 7.97 | 61.5 | 4.28 | 27.0 | 2.97/3.07 |  |
| **A302** | 123.2 | 8.21 | 54.8 | 4.13 | 17.9 | 1.48 |  |
| **R303** | 117.3 ^c^ | 7.95 | 58.4 | 4.10 | 30.0 | 1.86/1.86 | γ 27.2 (1.68/1.56), δ 43.2 (3.14/3.14), ε (7.21) |
| **Y304** | 118.9 ^c^ | 7.94 | 59.9 | 4.35 | 38.1 | 3.10/3.16 | δ 132.8/132.8 (7.11/7.11), ε 117.9/117.9 (6.82/6.82) |
| **R305** | 119.0 | 8.07 | 58.1 | 4.05 | 30.3 | 1.91/1.91 | γ 27.5 (1.64/1.84), δ 43.5 (3.20/3.20), ε (7.24) |
| **A306** | 121.9 | 7.96 | 53.8 | 4.20 | 18.4 | 1.47 |  |
| **K307** | 117.4 ^c^ | 7.94 | 57.4 | 4.18 | 32.6 | 1.85/1.85 | γ 24.6 (1.42/1.47), δ 29.0 (1.69/1.69), ε 42.0 (2.97/2.97) |
| **K308** | 119.8 | 8.04 | 56.9 | 4.16 | 32.4 | 1.74/1.71 | γ 24.4 (1.31/1.31), δ 28.7 (1.62/1.62), ε 41.9 (2.97/2.93) |
| **H309** | 117.9 | 8.13 | 56.1 | 4.57 | 29.5 | 3.27/3.16 | γ 132.4, δ2 120.4 (7.18), ε1 136.9 (8.23) |
| **A310** | 124.1 | 8.04 | 52.5 | 4.28 | 19.2 | 1.42 |  |

^a 1^H Chemical shifts are reported in ppm with an accuracy of ±0.02 ppm. ^13^C Chemical shifts are reported in ppm with an accuracy of ±0.1 ppm.

^b^ Carbon chemical shifts first (except when nitrogen chemical shift is referred, in that case it is indicated in the table), in brackets the proton chemical shift.

^c^ These signals may be interchangeable.
