# Supplementary material for: New Human Papilloma Virus E2 Transcription Factor Mimics: A Tripyrrole-Peptide Conjugate with Tight and Specific DNA-Recognition
Source: PLoS One. 2011 Jul 25;6(7):e22409. doi: 10.1371/journal.pone.0022409 (PMC3143144; doi:10.1371/journal.pone.0022409)
Supplement: Table S2 — 1H, 13C, and 15N chemical shifts assignments of the Dst moiety of αE2- conj . (DOCX) [file pone.0022409.s005.docx]

**Table S2.** ^1^H, ^13^C, and ^15^N Chemical shifts assignments of the *Dst* moiety of **αE2-*conj*** in a 4:6 TFE:aqueous solution (20 mM phosphate buffer pH 6.5, 2 mM DTT and 0.01% NaN_3_) at 25 °C and pH 6.5.^a,b^

| K296-NHζ | **1**-Me | **2**-NH_2_ | **3**-CH_2_ | **4**-CH_2_ | **5**-CH_2_ |
| --- | --- | --- | --- | --- | --- |
| n.d. (5.8) | 24.1 (2.04) | n.d. | 39.3 (2.92) | 28.0 (1.84) | 45.5 (3.32) |
| **7**-CH_2_ | **8**-CH_2_ | **9**-CH_2_ | **10**-CH_2_ | **11**-CH_2_ | **12**-CH |
| 49.1 (3.11) | 29.3 (1.52) | 25.7 (1.23) | 33.4 (1.74) | 50.7 (4.26) | 122.1 (7.15) |
| **14**-CH | **16**-NH | **18**-Me | **19**-NH | **21**-CH | **23**-CH |
| 108.6 (6.80) | 125.3 (9.49) | 24.6 (2.11) | 117.4 (9.38) | 123.2 (7.20) | 108.5 (6.87) |
| **24**-Me | **25**-NH | **27**-CH | **29**-CH | **30**-Me | **31**-NH |
| 38.3 (3.85) | 117.0 (9.40) | 123.1 (7.14) | 108.5 (6.85) | 38.3 (3.82) | 111.2 (7.93) |
| **32**-CH_2_ | **33**-CH_2_ | **34**-CH_2_ | **35**- and **36**-Me |  |  |
| 38.3 (3.42) | 27.2 (2.01) | 57.9 (3.14) | 45.2 (2.87) |  |  |

^a 1^H Chemical shifts are reported in ppm with an accuracy of ±0.02 ppm. ^13^C Chemical shifts are reported in ppm with an accuracy of ±0.1 ppm.

^b^ Carbon chemical shifts first (except when nitrogen chemical shift is referred, in that case it is indicated in the table), in brackets the proton chemical shift.
