# Supplementary material for: New Human Papilloma Virus E2 Transcription Factor Mimics: A Tripyrrole-Peptide Conjugate with Tight and Specific DNA-Recognition
Source: PLoS One. 2011 Jul 25;6(7):e22409. doi: 10.1371/journal.pone.0022409 (PMC3143144; doi:10.1371/journal.pone.0022409)
Supplement: Text S1 — αE2- conj purification. (DOC) [file pone.0022409.s006.doc]

**SUPPLEMENTARY TEXT**

**Text S1 . αE2-*conj* purification**

Cleavage/deprotection of the resin-bound hybrids under standard conditions afforded major products that were purified by preparative RP-HPLC (Vydac C18 column, gradient 5 to 40% B over 30 min, A: H_2_O 0.1% TFA, B: CH_3_CN 0.1% TFA,). Yield of conjugate **αE2-*conj*** was of ~20% considering peptide synthesis. Retention Time: 18.1 min. MS: MALDI-TOF (M+H^+^): Calcd. for C_121_H_200_N_43_O_29_S = 2753.2, Found 2752.9.
