# Supplementary material for: New Human Papilloma Virus E2 Transcription Factor Mimics: A Tripyrrole-Peptide Conjugate with Tight and Specific DNA-Recognition
Source: PLoS One. 2011 Jul 25;6(7):e22409. doi: 10.1371/journal.pone.0022409 (PMC3143144; doi:10.1371/journal.pone.0022409)
Supplement: Text S2 — NMR chemical shit assignments of αE2- conj . (DOC) [file pone.0022409.s007.doc]

**SUPPLEMENTARY TEXT**

**Text S2. NMR chemical shit assignments of αE2-*conj***

A variety of 2D NMR experiments were performed to allow the assignment of proton, carbon and nitrogen chemical shifts of **αE2-*conj*** (see **Figure S1** and **Tables S1** and **S2**). The strategy followed involved several 2D gradient-enhanced experiments such as [^1^H-^15^N] HSQC, [^1^H-^13^C] HMQC and [^1^H-^13^C] HMQC-TOCSY heteronuclear and [^1^H-^1^H] TOCSY and NOESY homonuclear experiments. First, we performed the NMR experiments of the peptide moiety alone (with an alanine in residue position 296, **αE2-*Ala***) to assign the peptide chemical shifts (see **Figure S1C** right panel). The experiments were carried out in a 4:6 TFE:aqueous solution. The aim was to induce some helical conformation in the peptide for observing a more dispersed set of resonances, thus facilitating the chemical shift assignment, and also for assisting to the solubility of **αE2-*conj*** at the concentrations required for the NMR experiments. The ^15^N chemical shift assignments were performed through a [^1^H-^15^N] HSQC experiment. However, in some cases, when overlapping of signals in the proton dimension was present in the amide region, it was not possible to determine which nitrogen chemical shift corresponded to which amino acid.

Then, we proceeded to analyze the hybrid that was dissolved in the same TFE solution (see **Figures S1B** and **S1C** left panel). Through the [^1^H-^13^C] HMQC-TOCSY it was possible to identify the spin system corresponding to positions **3**-**5**, **7**-**11** and **31**-**34**. The last spin system presents correlation peaks in both TOCSY spectra and in the NOESY spectrum with a signal at 7.93 ppm, the amidic proton **31**. Protons of position **5** show crosspeaks in the NOESY spectrum with protons **3**, **4**, **7**, **8** and K296-NHζ; while proton **3** only with protons **4** and **5**. The signals corresponding to the six methines in the pyrrole functions were determined from the aromatic [^1^H-^13^C] HMQC spectrum, appearing approximately at ~108.5 and ~6.80-6.85 ppm for ^13^C and ^1^H, respectively, for positions **14**, **23** and **29**, and at ~122-123 and ~7.14-7.20 ppm for positions **12**, **21** and **27**. The spin systems of the each pyrrole ring were determined from the homonuclear TOCSY experiment. In the low field region of the proton spectrum three signals were observed at 9.49, 9.40 and 9.38 ppm (125.3, 117.4 and 117.0 ppm are the respective chemical shifts for the ^15^N nuclei). The 9.49 ppm proton signal presented a crosspeak in the NOESY spectrum with a proton at 2.11 ppm. This crosspeak allowed us to identify the methyl of the acetylene function in position **18**, and this NH as corresponding to position **16**. The other methyl group at 24.1 and 2.04 ppm for ^13^C and ^1^H, respectively, corresponded to the N-terminal acetylated function in the peptide sequence (position **1**). The proton signal at 9.49 ppm presented other key crosspeak in the NOESY spectrum with a proton at 6.80 ppm, which allowed us to identify the signals corresponding to the pyrrole ring positions **12** and **14**. Additionally, position **12** showed NOESY cosspeaks with methylene protons corresponding to the spin system **7**-**11**. On the other hand, the proton signal at 9.38 ppm presented a NOE crosspeak with position **14** and with a proton at 6.87 ppm, thus assigned to **21**. Positions **23** and **21** presented a NOESY crosspeak with a methyl group at 3.85 ppm (position **24**). Analogously, we assigned the remaining pyrrole ring, whose position **29** (6.85 ppm) presented also a NOESY crosspeak with the amide proton of position **31** (7.93 ppm) and the methylene protons of position **32** (3.42 ppm). Amine protons were not observed in the NMR spectra due to the exchange with the solvent.

Finally, we determined that the connection between the *Dst* and peptide moieties in the conjugate was achieved by the secondary amine function in the linker, and not by the primary amine (**Figure S1A inset**). First, in the region of 5.00-6.50 ppm of the spectrum, corresponding to the NHs of urea functions, only one signal at 5.80 ppm was present. This signal showed correlation crosspeaks in the TOCSY spectra only with signals corresponding to the side chain of K296, allowing us to assign it as K296-NHζ. Second, signals corresponding to the spin system of positions **3**-**5** did not present correlation crosspeaks with any other proton in the TOCSY spectra. If the substitution in the linker were carried out by the primary amine function, it should be expected to observe an additional signal in the NH urea region and the corresponding correlation crosspeaks of this NH proton with protons of positions **3**-**5** in the TOCSY spectra. Unfortunately, the signal of the nitrogen bonded to proton at 5.80 ppm was not detected in the [^15^N-^1^H] HSQC experiment. Third, the signal at 5.80 ppm showed in the NOESY spectrum crosspeaks with protons of side chain of K296 and with protons of both **3**-**5** and **7**-**11** spin systems. In particular, it showed a strong NOESY crosspeak with proton of position **5** (at 3.32 ppm in a rather clean region of the proton spectrum) and a weaker one with protons of position **4**. On the contrary, this NH proton did not present a crosspeak with the signal at 2.92 ppm (position **3**), as it should be expected in the case of connection through the primary propylamine. Furthermore, a strong crosspeak was observed between this NH and protons of position **8** and a weaker one with protons of position **9** (signal of position **7** is overlapped with K296-Hεs). In the case of substitution through the propylamine, the K296-NHζ proton should present NOEs only with protons of the spin system **3**-**5**, especially with position **3** and not with protons of the spin system **7**-**11**. Finally, as mentioned before, protons of position **5** show cross-peaks in the NOESY spectrum with protons **3** and **4**, with protons of the spin system **7**-**11** and a strong cross peak with K296-NHζ; while proton **3** only correlated with protons **4** and **5**.The analysis the secondary chemical shifts in the **αE2-*conj*** peptide compared with the isolated **αE2** peptide is shown in **Figure S2**.
