# Supplementary material for: New Human Papilloma Virus E2 Transcription Factor Mimics: A Tripyrrole-Peptide Conjugate with Tight and Specific DNA-Recognition
Source: PLoS One. 2011 Jul 25;6(7):e22409. doi: 10.1371/journal.pone.0022409 (PMC3143144; doi:10.1371/journal.pone.0022409)
Supplement: Text S3 — TFE titrations. (DOC) [file pone.0022409.s008.doc]

**SUPPLEMENTARY TEXT**

**Text S3. TFE titrations**

A TFE titration of the peptides was performed in order to test wether **αE2-*Ala*** and **αE2-*conj*** have a different helical population in water solution respect to the isolated **αE2** peptide.

TFE titration experiments were done as previously described [10]. Briefly, 20 μM peptide solution in 10 mM phosphate pH 7.0, with increasing TFE concentrations were incubated overnight at room temperature before measuring ellipticity at 222 nm. CD measurements were carried out on a Jasco J-810 spectropolarimeter using a Peltier temperature-controlled sample holder at 25 ºC.

It is possible to consider that the free energy for α-helix depends linearly on the TFE/water molar ratio with a proportionality constant *m*:

$$\begin{matrix} \Delta G_{coil\leftrightarrow\alpha-helix}^{TFE}=\Delta G_{coil\leftrightarrow\alpha-helix}^{water}-m\cdot\frac{[TFE]}{[water]} \\ \end{matrix}$$

In that case, the molar ellipticity at 222 nm during the titration can be fitted to the following equation to extract values for *ΔG ^water^* and *m*:

$$\left[\theta\right]=\frac{\left[\theta\right]^{TFE}+\left[\theta\right]^{water}\cdot\exp\left(-\Delta G^{TFE}/RT\right)}{1+\exp\left(-\Delta G^{TFE}/RT\right)}$$

where [θ]^water^ and [θ]^TFE^ are the mean residue ellipticities in water and at high cosolvent concentration, R is the gas constant and T is the absolute temperature.

Molar ellipticity at 222 nm for **αE2**, **αE2-*ala*** and **αE2-*conj*** was well fitted with the same *ΔG^water^* (1.1± 0.2 kcal/mol), so we can conclude that the three peptides have a similar helical population (see **Figure S3)**.
